# Migration Activity of *Spodoptera litura* (Lepidoptera: Noctuidae) between China and the South-Southeast Asian Region

**DOI:** 10.3390/insects15050335

**Published:** 2024-05-06

**Authors:** Yifei Song, Xinzhu Cang, Wei He, Haowen Zhang, Kongming Wu

**Affiliations:** 1 Institute of Insect Sciences, College of Agriculture and Biotechnology, Zhejiang University, Hangzhou 310058, China; 2 State Key Laboratory for Biology of Plant Diseases and Insect Pests, Institute of Plant Protection, Chinese Academy of Agricultural Sciences, Beijing 100193, Chinamarszhang_0@163.com (H.Z.); 3 State Key Laboratory of Ecological Pest Control for Fujian and Taiwan Crops, Institute of Applied Ecology, Fujian Agriculture and Forestry University, Fuzhou 350002, China

**Keywords:** transboundary migration, movement ecology, pest outbreaks, trajectory simulation

## Abstract

**Simple Summary:**

The common cutworm, *Spodoptera litura*, is a major migratory pest worldwide. Seasonal migration is the biological basis of its regional population outbreaks. In this study, migration dynamics and trajectory simulations were conducted from Ruili City, an important migratory corridor between China and the south Asia region. The results showed that this pest exhibited seasonal migration in this area, mainly divided into two migration periods. One seasonal migration route across multiple national borders was identified by a trajectory simulation model. These findings will help in developing regional management systems for this pest in the relevant countries.

**Abstract:**

The common cutworm, *Spodoptera litura* (F.), feeds on a wide variety of food and cash crops and is one of the most widespread and destructive agricultural pests worldwide. Migration is the biological basis of its regional population outbreaks but the seasonal movement of this pest between east and south Asia regions remains unknown. In this study, searchlight traps were used to monitor the seasonal migration of *S. litura* from 2019 to 2023 in Ruili City (Yunnan, China), located along the insect migratory route between China and the south Asia region. The results showed that migratory activity could occur throughout the year, with the main periods found in spring (April–May) and autumn (October–December). The ovarian development and mating status of the trapped females indicated that most individuals were in the middle or late stages of migration and that Ruili City was located in the transit area of the long-distance migration of the pest. In the migration trajectory simulation, populations of *S. litura* moved from northeast India, Bangladesh, and northern Myanmar to southwestern China along the southern margin of the Himalayas in spring and returned to the south Asia region in autumn. Our findings clarify the seasonal migration patterns of *S. litura* in China and South Asia and facilitate the development of regional cross-border monitoring and management systems for this pest.

## 1. Introduction

The common cutworm, *Spodoptera litura* (Fabricius) (Lepidoptera: Noctuidae), is widely distributed throughout the tropical and subtropical areas of Asia and Oceania [1,2]. Larvae of *S. litura* are polyphagous and feed on more than 250 host plants, for example, pepper, eggplant, soybean, corn, cotton, and tobacco. Thus, *S. litura* is one of the most destructive herbivores worldwide [1,3]. In the Indian Peninsula, for example, *S. litura* has become the most damaging pest of vegetables and cotton, causing an annual yield loss of 10–30% [4]. In East Asia, *S. litura* primarily infects crops such as peanuts, soybeans, and cotton [1,5]. In most major crops, *S. litura* larvae are commonly treated with synthetic and biological insecticides. However, increasing levels of resistance to biocides present challenges in terms of control and result in economic losses for farmers [6,7].

*S. litura*, *Spodoptera exigua*, and *Spodoptera frugiperda* are the three major migratory pests of the *Spodoptera* genus and long-distance migration plays a key role in these pest outbreaks. In the 1970s, *S. litura* moths captured on a ship in the Pacific Ocean and the East China Sea were identified as migratory populations [8,9]. Additionally, the number of trapped moths at monitoring sites located more than 200 km away increases with the emergence of typhoons [10]. Tojo et al. demonstrated that this pest can migrate across the East China Sea to western Japan and South Korea from southern China during spring and early summer [11]. Long-term observations in Bohai Bay, China, have also confirmed the cross-sea migration of *S. litura* in northeast and central China [12]. In recent years, microsatellite and population genetic studies have indicated that this species migrates along the India–South China–Japan route and adapts to wide-ranging ecological conditions [13,14].

In east Asia, *S. litura* has between three and nine generations per year from north to south; its distribution can be divided into three regions: summer breeding areas, overwintering areas, and annual breeding areas [13,15]. In south and southeast Asian countries, *S. litura* can occur year-round, as observed in India, Myanmar, and Thailand [16,17]. Monsoon activity and various crop structures influence the temporal and spatial distributions of *S. litura* [18]. Previous studies mainly focus on the cross-sea migration of *S. litura* in East Asia and population genetics partly explain the movement ecology of this species. However, studies of the cross-border migration of *S. litura* remain scarce, particularly in east, south, and southeast Asia [11,12,13]. The western region of Yunnan province, China, is the geographical intersection between east Asia and southeast Asia bordering Bangladesh, India, and other South Asian countries. These unique geographical and climatic characteristics create notable migration routes for insects across multiple borders [19]. In this study, we monitored the long-term movement dynamics and simulated trajectories of *S. litura* over Ruili City, western Yunnan. These findings provide novel insights into the migration patterns of this species in Asia and could facilitate improvements in pest management strategies.

## 2. Materials and Methods

### 2.1. Study Site

The population monitoring of *S. litura* was conducted in Ruili City, Dehong Prefecture, China (23°98′ N, 97°83′ E, 760 m above sea level), which shares a direct border with Myanmar and is the nearest area in Yunnan Province to Bangladesh and the northeastern states of India. This area has a typical south subtropical monsoon climate, with an average annual temperature of approximately 21 °C. It is the main producing area of food crops, as well as tropical and subtropical cash crops, in Yunnan Province. In addition to the primary rotation mode of maize-rice-maize, farmers also practice cultivating cruciferous crops in winter and spring. It covers an annual planting area of 22,300 ha, representing 67% of the total vegetable area year-round [20,21]. Ruili experiences a warm southwest monsoon that prevails during spring and summer and is influenced by both the East Asian monsoon and the plateau monsoon in winter; thus, this migration channel is important for many migratory pests from southeast and south Asian countries that spread to China [22,23].

### 2.2. Searchlight Trapping Moth Method

The previous studies showed that the searchlight trap could effectively capture the migrating population of *S. litura* [5,12]. From 2019 to 2023, searchlight traps were used to capture *S. litura* moths, which effectively attracted flying insects at altitudes of up to 500 m. The light was equipped with a 1000 W metal halide bulb (JLZ1000BT; Shanghai Yaming Lighting Co., Ltd., Shanghai, China), producing a vertical beam of light with a luminous flux of 105,000 lm, color temperature of 4000 K, and color rendering index of 65 [24]. A lamp with a parabolic reflector was installed at the center of a metal funnel with a diameter of 1 m and a net bag was hung at the bottom of the funnel to collect the trapped insects. The monitoring period was from 17 January 2019 to 31 December 2023. The searchlight was turned on at sunset every day and off at sunrise the next day, except during extreme weather, power outages, or lamp damage. After 1 d of freezing treatment, the trapped insects were classified and counted based on morphological characteristics and the *S. litura* moths were stored in the refrigerator at −20 °C for additional experiments.

### 2.3. Ovarian Dissection Method for Trapping Female Moths

The ovarian development status of migratory pests can effectively reflect their population characteristics. Similar to *S. frugiperda*, local populations typically have lower ovary levels than immigrant populations, especially in migratory corridors [25]. In this study, the ovarian development status of *S. litura* was classified into five levels according to Zhao et al. and Wu et al.: the transparent opalescent phase (level 1), yolk deposition (level 2), egg maturation (level 3), the peak oviposition phase (level 4), and the terminal phase (level 5) [26]. The mating status was inferred by the presence of spermatophores in females. In total, 20 female moths were dissected daily (or all individuals if the total number of captures was less than 20) to examine their ovaries by using a stereoscopic dissecting microscope (TS-75X; Shanghai Shangguang New Optical Technology Co., Ltd., Shanghai, China).

### 2.4. Trajectory Simulation

#### 2.4.1. Peak Days of Population Migration

Based on the overall migratory curves of *S. litura* per year, the peak migratory days during 2019–2023 were identified as 26 April and 2 November in 2019 (2288 and 61 individuals, respectively), 16 May and 15 November in 2020 (596 and 46 individuals, respectively), 17 May and 11 October in 2021 (304 and 95 individuals, respectively), 27 April and 28 October in 2022 (275 and 307 individuals, respectively), and 15 April and 11 November in 2023 (725 and 234 individuals, respectively).

#### 2.4.2. Meteorological Data and Trajectory Simulation Model

For trajectory simulation, meteorological datasets from the Final Analysis Date (FNL) were obtained from the National Centers for Environmental Prediction and the National Center for Atmospheric Research. These data were used to initialize the meteorological field data and boundary conditions of the Weather Research and Forecasting model (WRF). A 10 km × 10 km grid-spaced meteorological dataset was output once per hour as the background condition for calculating insect migration paths after the FNL input WRF model simulation. The simulation date was the peak day of population migration. The central position was the searchlight trap, described in a prior section. In this study, a single-layer nested grid design method based on InsectTrace-WRF was adopted [27]. Table 1 presents the specific model scheme based on Wu et al. [23,28].

The trajectory simulations were conducted according to the migratory biology of moths. (i) The moths are used to take off at dust with a peak time of 20 min after sunset and land at the dawn of the next day with a peak time of 10 min before sunrise [29,30]. In this study, the start times of the trajectory simulation as well as the end times in spring and autumn were set according to the seasonal change (https://sunrise.maplogs.com/zh-CN/ruili_dehong_yunnan_china.279998.html (accessed on 4 May 2024). For example, the start and end times of the migratory simulation on 26 April 2019 were 20:04 and 06:38 for spring migration, while the start and end times on 2 November 2019 were set to 18:59 and 07:15 for autumn migration, respectively. (ii) Radar observations have shown that moths primarily fly in the high altitudes of 100 to 1000 m above the ground and their movement direction aligns with the wind direction [11,12]. (iii) The flight altitudes of *S. litura* moths were set at 100, 200, 300, 400, 500, 600, 700, 800, 900, and 1000 m above ground level (AGL) for covering their most probable flight altitudes. Because moths may land at any time after taking off, the simulation coordinates at each flight altitude are estimated at 10 min intervals using the WRF model. Therefore, a migration trajectory consisting of continuous points was obtained, with the latest one as an endpoint of the simulation [11,31]. (iv) Many migratory pests begin to migrate only when the ambient temperature exceeds the critical temperature, such as *Loxostege sticticalis* and *Mythimna separata* [32,33]. In this study, we adopted the temperature threshold (13.1 °C) of *S. frugiperda* because of the absence of relevant research on *S. litura* and their highly similar biology [34]. ArcGIS Pro 3.0 (Esri, Redlands, CA, USA) was used to visualize the migration trajectory.

### 2.5. Statistical Analysis

Differences in mating status, ovarian development level, and number of trapped moths across different years and months were analyzed using a two-way analysis of variance and then Tukey’s HSD test (year and month as fixed factors). The chi-square test was used to compare the male-to-female ratio for each month, with a theoretical sex ratio of 1:1. All data were checked using the Shapiro–Wilk test for normality and Levene’s test for homogeneity of variance. The percentage data were subjected to an arcsine square root transformation. All statistical analyses were performed using SPSS (version 26.0; IBM, Armonk, NY, USA). The daily number of trapped moths from 2019 to 2023 was grouped using the optimal partitioning of ordered samples in the Data Processing System V9.50 and the migration period of *S. litura* was accordingly divided [35].

## 3. Results

### 3.1. Ovarian Development Dynamics of Female Spodoptera litura

**Figure 1:**
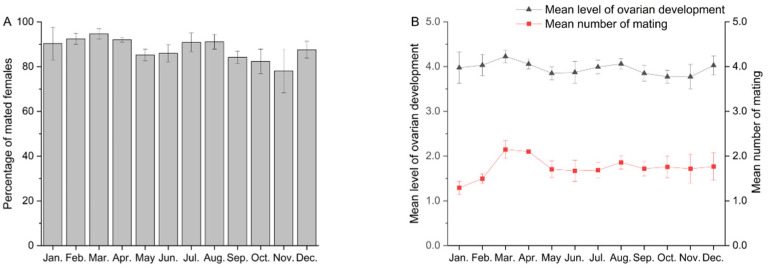
Ovarian development status of trapped *Spodoptera litura* females during each month from 2019 to 2023. (**A**) Percentage of mated females. (**B**) Mean levels of ovarian development and number of mating.

A total of 70,835 *S. litura* moths were captured over the five years. From 2019 to 2023, most *S. litura* females exhibited high reproductive development, characterized by increased levels of ovarian development, mating rates, and mating frequency (Figure 1). Specifically, the mating rate of females was less than 80% only in November, while reaching the highest mating rate, 94.7%, in March (Figure 1A). Analysis of variance revealed that the mating rate of *S. litura* varied significantly across years and that the monthly differences were not statistically significant (*F* = 9.022, *df* = 4, *p* < 0.001; *F* = 1.620, *df* = 11, *p* = 0.131). The average level of ovarian development in the captured females was over 3.7 and the mating frequency exceeded 1.5. The highest values recorded were 4.2 and 2.2 in March (Figure 1B). Ovarian development and mating status showed significant differences among years (Ovary: *F* = 3.298, *df* = 4, *p* = 0.020; Mating: *F* = 4.420, *df* = 4, *p* = 0.005). Females mating numbers change significantly monthly but those for ovarian development do not (Ovary: *F* = 1.517, *df* = 11, *p* = 0.165; Mating: *F* = 3.493, *df* = 11, *p* = 0.002). These results indicate that the trapped *S. litura* individuals were in the middle to late stages of migration.

### 3.2. Seasonal Population Migration Dynamics of Spodoptera litura

**Figure 2:**
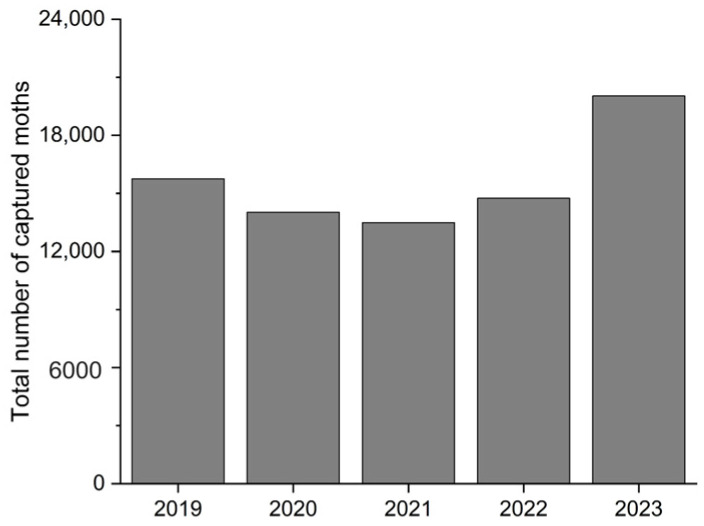
Annual catches of *Spodoptera litura*, 2019–2023.

The number of annual catches initially decreased and then increased, with the highest number being 20,029, recorded in 2023 (Figure 2). Two-way ANOVA showed that the year did not have a significant effect on nightly catches (*F* = 0.763, *df* = 4, *p* = 0.555). However, the number of trapped moths in April and May was significantly higher than those in other months (*F* = 10.114, *df* = 11, *p* < 0.001). The highest average catches of *S. litura* per day occurred in April and May, with both months exceeding 100 individuals and catches in the other months were relatively low, especially in winter. Additionally, the percentage of females initially increased and then decreased but the annual catch of females was much lower than that of males overall (Table 2). Specifically, the highest female rate was 60.4% in April, the only month when female catches exceeded those of males (*x*^2^ = 184.9, *p* < 0.001), and in other months, it was less than 50%. The effect of the month on the *S. litura* female proportion was significant; it was higher in April than that in other months (*F* = 12.485, *df* = 11, *p* < 0.001). Notably, higher monthly catches were associated with higher proportions of females (Table 2). 

**Figure 3:**
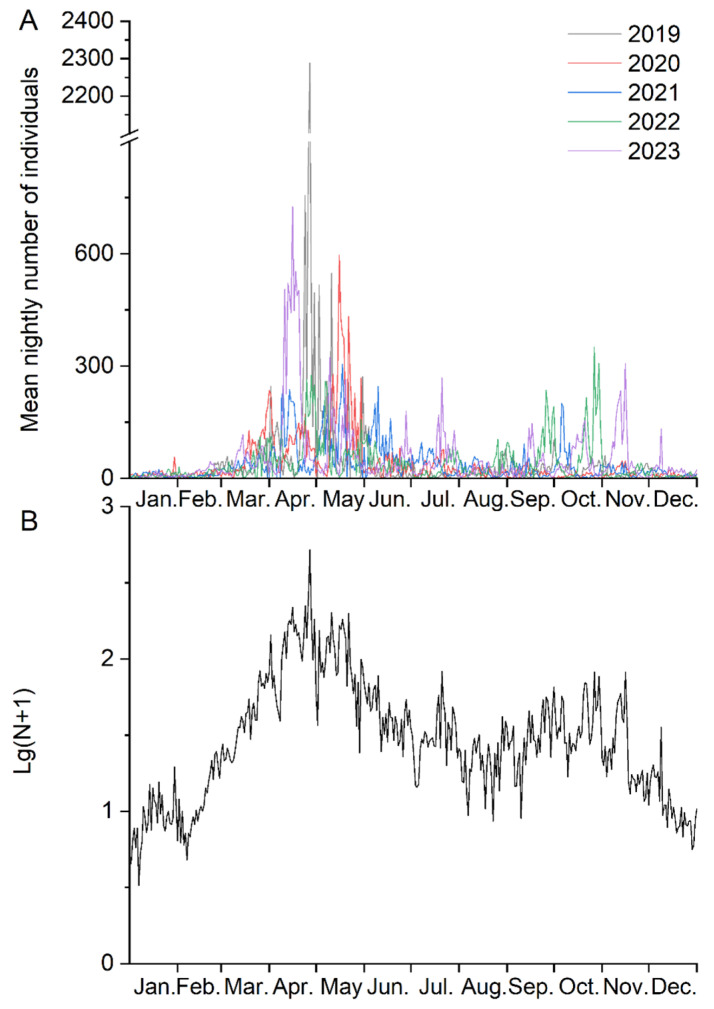
Nightly catches (**A**) and mean logarithm numbers (**B**) of the *Spodoptera litura* moths captured in light traps, 2019–2023.

Based on the number of monthly catches, the entire year was divided into four periods by using the optimal segmentation of ordered samples: January–March, April–May, June–September, and October–December. This result not only has the lowest error function but also reasonably reflects the seasonal migratory population (Table 3). Therefore, in this study, we considered April and May as the peak spring migration period, October to December as the autumn migration period, and the remaining months as the transition period (Figure 3A). This result showed a consistent stable seasonal migration activity over the past 5 years (Figure 3B).

### 3.3. Trajectory Simulation and Ovarian Development of Spodoptera litura Populations in Peak Days

**Figure 4:**
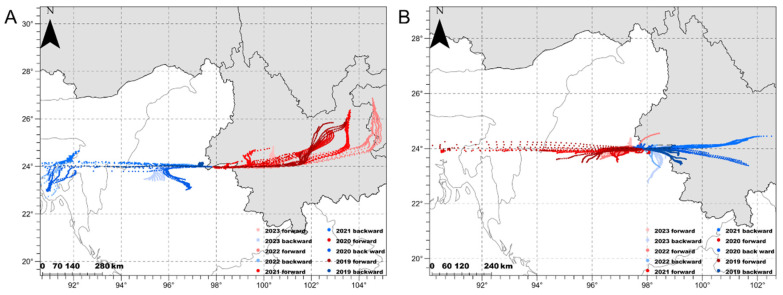
Simulated migration trajectories of *Spodoptera litura* moths in spring (**A**) and autumn (**B**).

**Figure 5:**
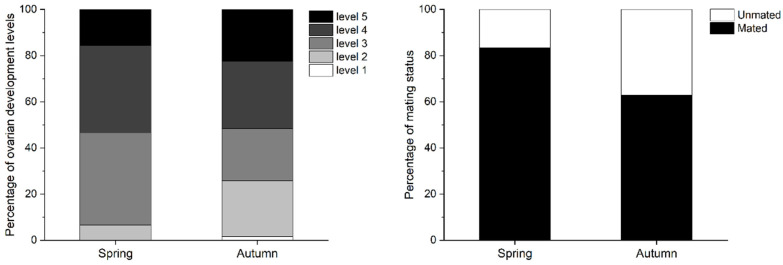
Ovarian development status of trapped *Spodoptera litura* females in peak days during the spring and autumn migration periods.

Simulation of the migration trajectory over 12 h on peak days revealed that the seasonal migration path of *S. litura* in China, Myanmar, Northeast India, and Bangladesh was centered at approximately 24° N. Generally, the spring migration period for colonization was longer than that of autumn, with the longest distance being close to 700 km. However, interactions between the populations of China and Burma were more than those of Bangladesh and Bangladesh (Figure 4; Table 4). Furthermore, the mating rate and ovarian development of *S. litura* were high during the spring and autumn migratory periods from 2019 to 2023 (Figure 5). Although most of the females trapped showed a high level of ovarian development, the percentage of matured females (ovarian level > 2) in the spring period (92.3%) was higher than those in autumn (74.2%). Correspondingly, the percentage of mated females (83.3%) was also higher than that in the autumn (62.9%).

During the spring migration of 2023, all take-off points of the backward trajectories were located in low-altitude areas in north-central Myanmar and Yunnan; in other years, they were also found in Bangladesh and India. Overall, 54.3% of the backward points were in Myanmar and 39.7% were in South Asia (i.e., Bangladesh and the northeastern State of India), which were the primary sources of *S. litura* in Ruili. By contrast, the majority of forward landing points were distributed in the central and northeastern regions of Yunnan Province. Compared with the spring migration period, the direction of autumn movement was opposite to that of spring. Interactions between *S. litura* populations in Myanmar and China were more frequent in autumn. In 2019 and 2021, the forward landing points were located in South Asian countries. Specifically, 95.3% of forward points were from China and Myanmar and 4.7% were from south Asian countries. For the backward simulation, all points were located in China and Myanmar, accounting for 48.1% and 51.9%, respectively (Figure 4; Table 4). In these figures, some unnatural longitudinal dots along the outer boundary of the plotted area were caused by the trajectory-stopping calculation after exceeding the simulation boundary.

## 4. Discussion

Regional population outbreaks are the primary factors hindering the prompt control of migratory pests during crop production. Therefore, timely effective monitoring and early warnings of pest migration are particularly crucial [36,37]. Yunnan is located on the border between east and southeast Asia. The unique geographical and climatic characteristics of the area provide a suitable migration route for major crop pests such as *S. litura* [19,38]. In this study, long-term observation of the seasonal pattern of migratory *S. litura* populations was conducted using searchlight trapping in Ruili, Yunnan, and the migration trajectory for peak days was simulated. First, the developed ovarian status of trapped females from 2019–2023 indicates that this species was largely migratory. Second, the population dynamics of the pest exhibited seasonal migration in this area, mainly divided into the spring-summer (April–May) and autumn (October–December) migration periods. Finally, a seasonal migration route across multiple national borders was simulated using a trajectory analysis based on the WRF model.

*S. litura*, a major migratory pest native to Asia, has garnered broad consensus but few studies have focused on its regional migratory patterns, especially in south or southeast Asia, over the past decades [11,12]. However, the seasonal migration of this pest has frequently been observed in the Sino-Burmese border. The migratory activity of insects often provides biological benefits [39]. For example, long-term migration studies in the Bohai Bay of China showed that the biomass of most migratory insects heading south (autumn migration) was 1–10 times higher than those heading north (spring and summer migration). This result revealed that cross-sea migration provides biological benefits to insect populations [40,41]. In this study, the population of *S. litura* during the spring migration period was higher than that during autumn. Because of the stable interannual population dynamics, variances in geographical and climatic characteristics may be related.

As an obligatory migrator, *S. litura* initiates movement during the immature stage of ovarian development to accelerate its reproductive development and generate alternation through flight [42]. In most cases, migratory moths are used to initiate flight in the immature stage of ovarian development and finish migration in the mature stage of ovarian development. The mature ovaries of the trapped *S. litura* females indicated that the populations originated from areas distant from Ruili. Additionally, this finding suggests that the populations engage in mating activities during migration, which facilitates the direct entry of pests into the oviposition stage and leads to outbreaks in the field after landing. Studies have shown that female and male *S. litura* exhibit strong and similar flight abilities and that mature females are capable of flying long distances [43,44,45]. The number of trapped individuals and the proportion of females from April to May were significantly higher than those in the other months, which was the main driver of their eastward migration in spring. This is important for subsequent colonization. Similar dynamics have been observed for *Helicoverpa armigera* and *S. litura* in Bohai Bay [12,46]. By contrast, the ovarian development of *S. litura* in Ruili consistently remained at a high level and in North China, during the autumn migration season, it was lower than that in Ruili. A probable explanation for this is that *S. litura* has the potential to colonize Yunnan and its surrounding areas. Therefore, it can infest host plants as soon as it finds them, eliminating the need to inhibit ovarian development and conserve the energy required for long-distance migration [47]. However, more energy needs to be allocated to flights migrating to South China.

Insect migration often relies on air currents and may span multiple generations. Consequently, the movement biology of insects varies in spatial and temporal patterns [48,49]. The western Yunnan region is adjacent to the Qinghai-Tibet Plateau but its altitude is relatively low. This region is influenced by the vigorous southwest monsoon in spring and summer and the east Asian monsoon and plateau monsoon in autumn and winter, which create favorable currents for the seasonal migration of *S. litura* [23,50]. The prevailing winds over Yunnan begin to shift in April each year and the warm and humid currents in the Bay of Bengal become the primary carriers of the eastward expansion of insects in south and southeast Asia by May [23,27]. The dynamics of *S. litura* populations in Ruili confirmed that the highest annual migration was between mid-April and mid-May. Trajectory simulations also showed that pests from Bangladesh and Myanmar were the main sources in the central and northeastern regions of Yunnan province in spring. Variations in temperature and air current strength may be related to the further migration distance during the spring migration period. When night temperature is suitable, insects maximize their migration distance by selecting an appropriate air current layer [48]. 

The relatively consistent synoptic field may be the primary factor contributing to the uniform trajectories in west–east directions (Supplementary Materials). Considering the mountainous terrain of Ruili city, when the mountains are perpendicular to the airflow, the airflow speed decreases, causing it to ascend or flow to both sides of the mountain. When the orientation of the mountains aligns with the airflow direction, the canyon effect will enhance the airflow speed between the canyons. Hence, there is a possibility of crossover in the simulation trajectory. Wu et al. (2019) also studied the wind and temperature fields at 925 hPa in southeast Asia and China. It can reflect the relationship between migration routes and the environment at lower altitudes [23,28].

Simulating insect migration trajectories can objectively reflect migration paths and provide valuable information for predicting and managing pests [50]. The WRF model, a new generation of high-resolution mesoscale numerical prediction systems that combines insect biological parameters, has been widely used in China [51]. Since the invasion of *S. frugiperda*, Wu et al. (2019) and Li et al. (2020) have successively predicted the invasion and spread of *S. frugiperda* by using the WRF model, which plays a crucial role in the early warning and control of this pest in China [23,52]. According to monitoring and trajectory analysis of pest populations, using Bt plants and food (sexual) traps along the migratory route can effectively decrease pest infestation. In this study, because we did not include the flight speed and direction of *S. litura*, the actual migration routes may differ [18,53,54]. In addition, the effective trapping height of the searchlight is about 500 m, which aligns with the typical flight height of insects monitored by insect radar in most cases (200–1000 m AGL; this is also the majority of the heights in our simulation) [48]. In general, autumn temperatures are quite low but the season is used to become an inversion layer that is suitable for insect migration. Previous studies have shown that insects flighted at higher altitudes in inversion layer in autumn. For example, the insect radar monitoring confirmed that *H. armigera* migrated up to 2000 m AGL in autumn and only 200–300 m AGL in summer [55,56]. Simulations at different altitudes can offer more information about insect migration routes in the natural environment.

In recent decades, the integrated development of global agricultural trade and climate change has aggravated disease and pest outbreaks [57,58]. Thus, major invasive pests are increasing more frequently (e.g., *S. frugiperda* and *Tuta absoluta*), while the prevalence and variety of indigenous pests are increasing as well [59,60]. Owing to the migratory habits of major pests and the common occurrence of cross-border colonization in Asia, establishing a regional management system in the relevant countries is necessary [61]. In this regard, a regional management strategy for the “Three Zones and Four Belts” (i.e., various control measures to reduce the migratory populations in both annual breeding areas and migratory transition zones as well as ecological regulations in main immigratory areas) has helped to decrease crop yield losses caused by *S. frugiperda* in China, setting a positive example for the sustainable management of *S. litura*.

## Figures and Tables

**Table 1:** The scheme and parameters of the WRF model.

Item	Domain
The number of grid points	200 × 200
Layers	32.0
Map projection	Lambert
Microphysics scheme	Thompson
Longwave radiation scheme	RRTMG
Shortwave radiation scheme	RRTMG
Surface layer scheme	Monin-Obukhov
Land/water surface scheme	Noah
Planetary boundary layer scheme	Mellor-Yamada-Janjic
Cumulus parameterization	Tiedtke

**Table 2:** Mean number and percentage of trapped *Spodoptera litura* females monthly, 2019–2023.

Month	Individuals	Percentage	*x* ^2^	*p*
Jan.	50.0 ± 21.4 b	21.5 ± 1.6 c	75.918	<0.001
Feb.	83.6 ± 49.1 b	25.8 ± 6.3 c	75.843	<0.001
Mar.	515.0 ± 199.1 b	38.7 ± 7.0 b	67.669	<0.001
Apr.	2570.8 ± 1795.3 a	60.4 ± 10.6 a	184.905	<0.001
May	1380.8 ± 634.5 a	42.3 ± 9.6 b	76.640	<0.001
Jun.	370.8 ± 201.3 b	28.9 ± 6.1 bc	228.122	<0.001
Jul.	287.8 ± 148.6 b	29.1 ± 6.3 bc	172.466	<0.001
Aug.	170.2 ± 52.5 b	25.7 ± 2.8 c	156.622	<0.001
Sep.	242.6 ± 187.8 b	25.8 ± 5.4 c	220.005	<0.001
Oct.	344.2 ± 296.9 b	28.3 ± 4.3 c	230.624	<0.001
Nov	210.2 ± 198.0 b	27 ± 5.4 b c	166.154	<0.001
Dec.	70.0 ± 28.6 b	21.2 ± 7.1 c	109.394	<0.001
Total	6296.0 ± 1914.1	39.9 ± 8.3	593.951	<0.001

Different lowercase letters within the same column indicate a statistically significant difference in the number (rate) of trapped females in different months (*p* < 0.05). The numbers of females and males were compared by month for significant differences by using a chi-square test.

**Table 3:** Optimal segmentation of monthly catches by searchlight trap, 2019–2023.

Divide Number	Optimal Segmentation	*df*_1_, *df*_2_	Error Function
2	January–June; July–December	1, 11	1.1393
3	January–March; April–May; June–December	2, 10	0.3685
4	January–March; April–May; June–September; October–December	3, 9	0.1911

**Table 4:** The percentage of landing or take-off points of simulated trajectories in seasonal migratory periods, 2019–2023.

Country	Backward		Forward	
	Spring	Autumn	Spring	Autumn
China	5.9	48.1	94.0	22.0
Myanmar	54.3	51.9	6.0	73.3
India	6.1	0	0	2.0
Bangladesh	33.6	0	0	2.7

## Data Availability

The datasets used and/or analyzed during the current study are available from the corresponding author upon reasonable request.

## Supplementary Materials

The following supporting information can be downloaded at https://www.mdpi.com/article/10.3390/insects15050335/s1. Figure S1: The air currents status during the peak days in spring migration periods; Figure S2: The air currents status during the peak days in autumn migration periods; Figure S3: The temperature status during the peak days in autumn migration periods; Figure S4: The temperature status during the peak days in autumn migration periods.
