# Supplementary material for: Migration Activity of Spodoptera litura (Lepidoptera: Noctuidae) between China and the South-Southeast Asian Region
Source: Insects. 2024 May 6;15(5):335. doi: 10.3390/insects15050335 (PMC11121980; doi:10.3390/insects15050335)
Supplement: Supplementary file 1 [file insects-15-00335-s001.zip › insects-2945504-supplementary.pdf]

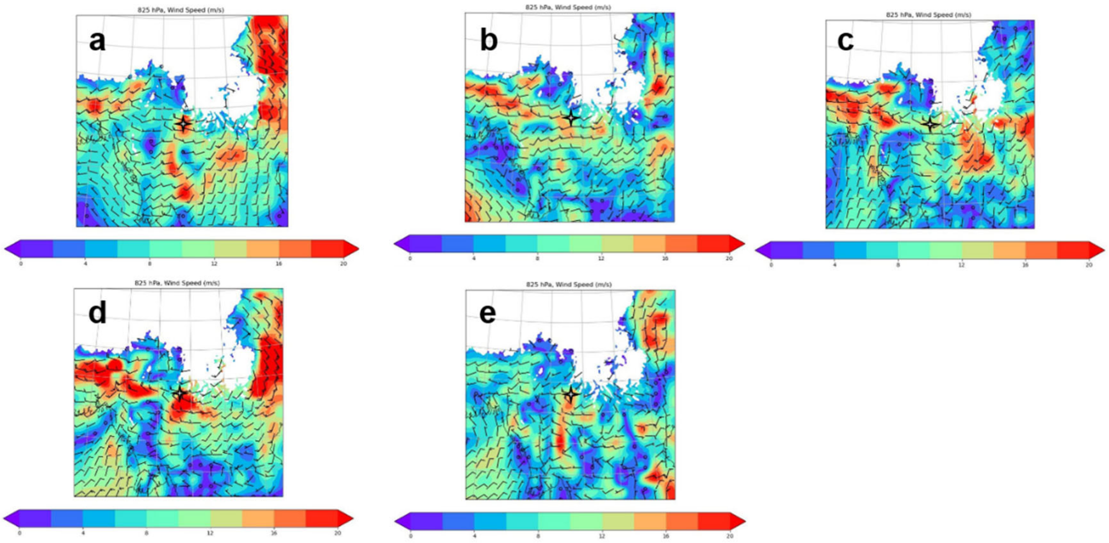

Figure S1. The air currents status during the peak days in spring migration periods. The star in the center of the map indicates Ruili City; a-e: 2019-2023.

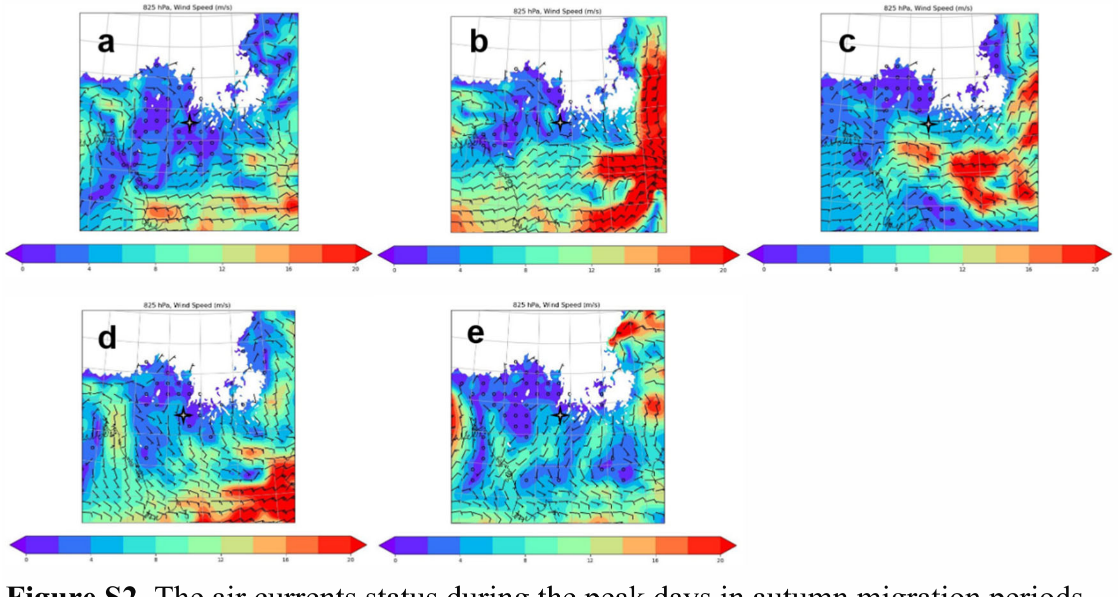

Figure S2. The air currents status during the peak days in autumn migration periods. The star in the center of the map indicates Ruili City; a-e: 2019-2023.

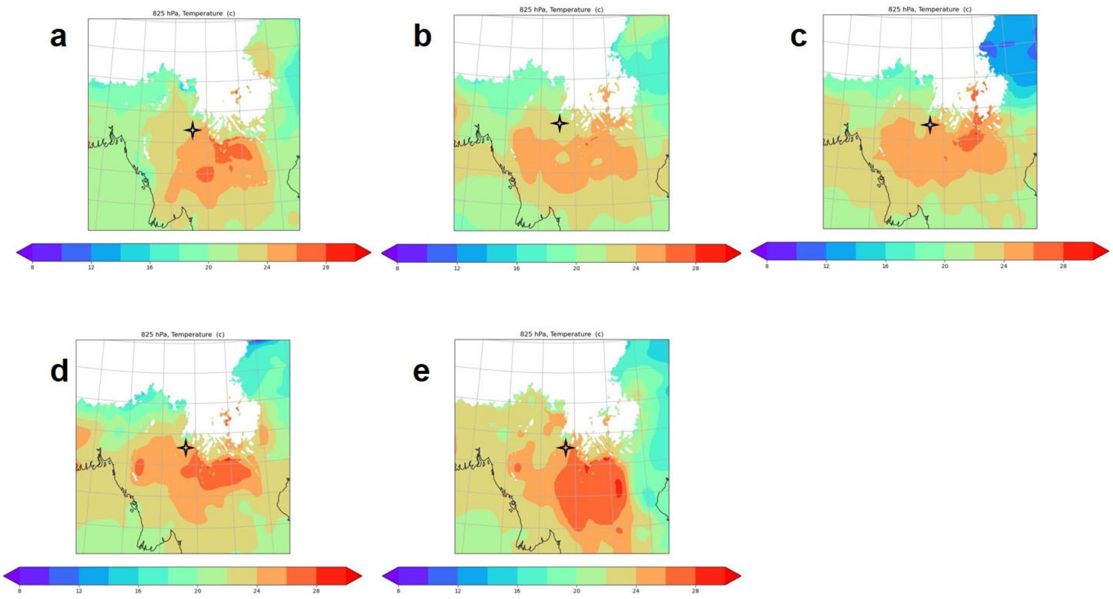

Figure S3. The temperature status during the peak days in autumn migration periods. The star in the center of the map indicates Ruili City; a-e: 2019-2023.

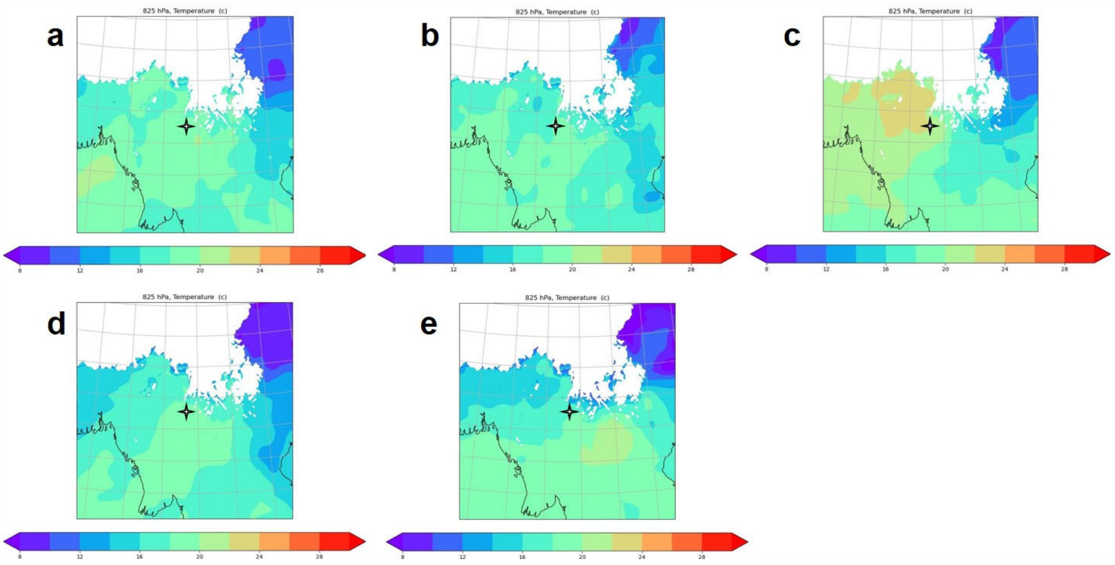

Figure S4. The temperature status during the peak days in autumn migration periods. The star in the center of the map indicates Ruili City; a-e: 2019-2023.
